# Association between weight-adjusted-waist index with hepatic steatosis and liver fibrosis: a nationally representative cross-sectional study from NHANES 2017 to 2020

**DOI:** 10.3389/fendo.2023.1159055

**Published:** 2023-05-19

**Authors:** Yun Shen, Yahui Wu, Minghan Fu, Kai Zhu, Jinsheng Wang

**Affiliations:** ^1^ Department of Pathology, Heping Hospital Affiliated to Changzhi Medical College, Changzhi, Shanxi, China; ^2^ Department of Pathology, Changzhi Medical College the First Clinical College, Changzhi, Shanxi, China; ^3^ Key Laboratory of Esophageal Cancer Basic Research and Clinical Transformation, Heping Hospital Affiliated to Changzhi Medical College, Changzhi, Shanxi, China

**Keywords:** WWI, steatosis, and fibrosis weight-adjusted-waist index, hepatic steatosis, liver fibrosis, NAFLD, NHANES, VCTE

## Abstract

**Background:**

The negative effects of obesity on hepatic steatosis and fibrosis have received considerable attention in recent years. The weight-adjusted-waist index (WWI) reflects weight-independent centripetal obesity. Herein, we provide the first investigation of a link between WWI, hepatic steatosis, and liver fibrosis.

**Methods:**

We used data from the National Health and Nutrition Examination Survey 2017-2020 to conduct a cross-sectional study. The linear relationship between WWI, controlled attenuation parameters, and liver stiffness measurements (LSM) was investigated using multivariate linear regression models. The nonlinear relationship was described using fitted smoothed curves and threshold effect analyses. Subgroup analyses were performed based on gender, age, body mass index, diabetes, hypertension, drinking, and smoking.

**Results:**

This population-based study included 7,594 people, 50.74% of whom were men and 49.26% of whom were women. Multivariate linear regression analysis revealed a significant positive relationship between WWI and hepatic steatosis [CAP, β=7.60, 95% confidence interval (CI) (4.42, 10.78), P<0.0001]. This positive association was stronger when excessive alcohol intake was present compared to when it was absent (P for interaction = 0.031), and when hypertension was present compared to when it was not (P for interaction = 0.014). The linear relationship between WWI and liver fibrosis was not statistically significant on multiple regression analysis [LSM, β=0.03, 95% CI (-0.26, 0.32), P=0.84]. However, a U-shaped association was seen between WWI and LSM, with a negative correlation when WWI< 10.92 and a positive correlation when WWI > 10.92.

**Conclusion:**

We report a strong association between WWI and hepatic steatosis, and suggest that it may potentially be used as a simple anthropometric index to predict hepatic steatosis.

## 1 Introduction

Non-alcoholic fatty liver disease (NAFLD) is the most common chronic liver disease worldwide, which affects up to 40% of adults and children (1, 2). NAFLD can progress from simple hepatic steatosis to non-alcoholic steatohepatitis (NASH) and then to liver fibrosis, cirrhosis, and hepatocellular carcinoma (3, 4). NAFLD has been linked to obesity and obesity-related metabolic disorders such as glucose intolerance, type 2 diabetes (T2D), and dyslipidemia (5).

Excessive hepatic fat accumulation not only leads to local changes, such as hepatocyte dysfunction, proinflammatory immune response activation, and fibrogenesis, but also triggers a series of extrahepatic metabolic disorders, including cardiovascular events and T2D. Furthermore, observational studies have highlighted that both hepatic steatosis and fibrosis are associated with a higher risk of all-cause mortality. Therefore, determining the level of liver steatosis is critical in the evaluation and clinical prognosis of patients with NAFLD (6). Although pathological biopsy remains the gold standard for evaluating the severity of hepatic steatosis and liver fibrosis, vibration controlled transient elastography (VCTE) is a non-invasive alternative that is increasingly being used. Recent observational studies have suggested that VCTE has robust accuracy in estimating the grade of hepatic steatosis and the stage of liver fibrosis (7, 8). However, the use of VCTE is limited by its popularity and high learning curve (9).

Weight-adjusted-waist index (WWI) was first postulated in 2018 as an anthropometric measure of central obesity that reflects both fat and muscle mass components, regardless of the body mass index (BMI) (10, 11). The links between WWI and various cardiovascular events have since been well established (11–14). However, the relationship between WWI and these hepatic indicators has not been defined.

In the present study, we used the National Health and Nutrition Examination Survey (NHANES) to investigate the relationship between WWI and hepatic steatosis and liver fibrosis in the US population.

## 2 Materials and methods

### 2.1 Data and sample sources

The NHANES is a cross-sectional survey conducted every two years to assess the nutritional and physical health of the general public in the United States (15, 16). Through interviews and related tests, demographics, dietary, and health-related information are collected (12, 13). The survey is approved by the Center for Disease Control and Prevention Research Ethics Review Board, and all survey participants provide written informed consent to participate (17).

**Figure 1:**
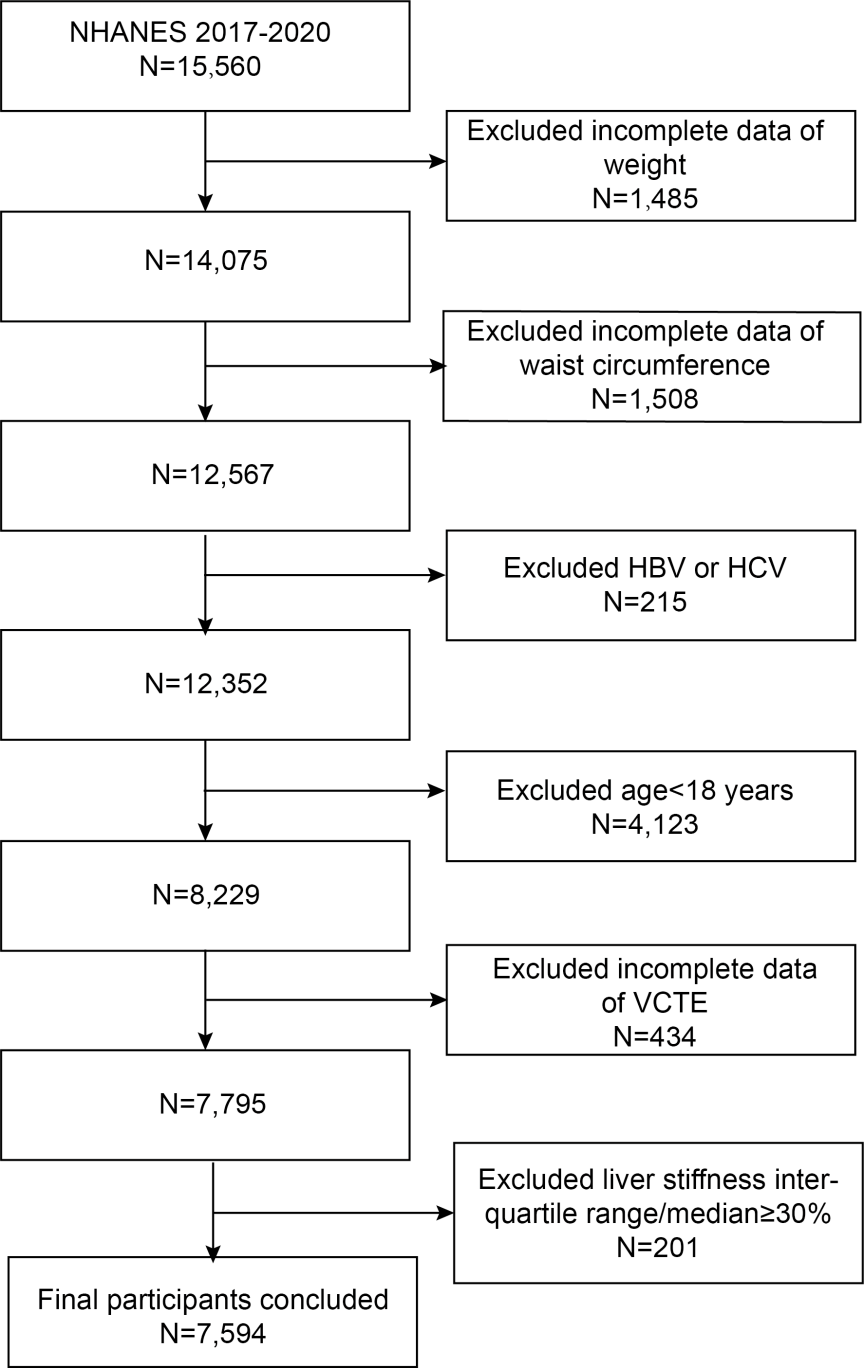
Flowchart of the sample selection from NHANES 2017–2020. NHANES, National Health and Nutrition Examination Survey; HBV/HCV, participants with infection of hepatitis B virus and hepatitis C virus; VCTE, vibration controlled transient elastography.

In the present study, we used the 2017–2020 pre-coronavirus-19 pandemic data from the NHANES database. Out of the 15,560 individuals who participated, we excluded those with hepatitis B or C infection (n=215), missing VCTE data (n=434), unreliable VCTE estimation (liver stiffness interquartile range/median≥30%, n = 201), those younger than 18 years old (n=4,123), and incomplete data on weight and waist circumference (WC) (n=2993). The final analyses included 7,594 participants (**Figure 1**).

### 2.2 Definition of weight-adjusted-waist index

WWI is a novel index that estimates central obesity based on WC and weight. WC and weight were measured in a mobile examination center (MEC), where laboratory tests were carried out under controlled conditions (14). WWI was included as an exposure variable in our study and calculated as follows:


$$\text{WWI}\left(\text{cm}/{\text{kg}}^{2}\right)=\text{WC}/{\text{Weight}}^{2}$$

### 2.3 Measurement of hepatic steatosis and liver fibrosis

Hepatic steatosis and liver fibrosis was detected with VCTE. Specifically, controlled attenuation parameter (CAP) and liver stiffness measurement (LSM) represented the levels of steatosis and fibrosis, respectively. We only included participants with a reliable VCTE estimation (interquartile range/median of LSM ≤ 30%).

### 2.4 Covariates

Based on a review of the literature, we summarized potential confounding covariates between WWI and hepatic steatosis and liver fibrosis in our multivariable-adjusted model (10, 11, 18, 19). Gender, age, race, family income-to-poverty ratio, and education level were all demographic covariates in our study. Anthropometric and laboratory covariates included BMI, direct high-density lipoprotein cholesterol (HDL, mmol/L), low-density lipoprotein cholesterol (LDL, mmol/L), triglycerides (TG, mmol/L), total cholesterol (TC, mmol/L), serum uric acid (μmol/L), alanine aminotransferase (ALT, IU/L), alkaline phosphatase (ALP, IU/L), and aspartate aminotransferase (AST, IU/L). Medical history covariates included the presence or absence of T2D, excess alcohol consumption (≥ 4 drinks per day), smoking, or cardiovascular disease (CVD, defined as history of coronary artery disease, congestive heart failure, heart attack, stroke, or angina pectoris).

### 2.5 Statistical analysis

Weighted Student’s t-tests for continuous variables and weighted chi-squared tests for categorical variables were used to assess differences between WWI quartiles. The NHANES used an inferential statistics method to represent a large, nationally representative sample due to its complex multistage probability sampling design. Thus, using linear regression analyses, we summarized continuous variables as means with standard errors (SE) and categorical parameters as proportions using logistic regression analyses. Weighted multivariable regression models were used in three different models to investigate the relationship between WWI and hepatic steatosis and liver fibrosis. No covariates were adjusted in Model 1. Model 2 was adjusted for gender, age, and race. Model 3 was adjusted for gender, age, race, education level, BMI, excess alcohol consumption, HDL, LDL, TG, total cholesterol, serum uric acid, ALT, ALP, AST, CVD, and T2D status.

We performed a sensitivity analysis after categorizing the WWI into quartiles to assess robustness. A generalized additive model (GAM) and smooth curve fitting were used to address non-linearity. When a non-linear correlation was observed, a two-piecewise linear regression model (segmented regression model) was used to fit each interval and calculate the threshold effect. Subgroup analyses were then performed based on gender, age, BMI, T2D, hypertension, excess alcohol consumption, smoking, and CVD. A log-likelihood ratio test was used to determine whether a threshold existed by comparing a one-line model (non-segmented) to a two-piecewise linear regression model. The inflection point (K) was determined using a two-step recursive method (14). Furthermore, a subgroup analysis of the correlations between WWI and hepatic steatosis and liver fibrosis was carried out using stratified multivariable logistic regression models with stratified covariates such as gender, age, BMI, and T2D. A two-sided P value ≤ 0.05 was considered statistically significant. R (version 4.1.3) and EmpowerStats, two statistical computing and graphical programs, were used to conduct the statistical studies (version 2.0).

## 3 Results

### 3.1 Baseline characteristics

**Table 1** summarizes the demographic profiles of the 7,594 participants. These participants had a mean ± SD age of 42.59 ± 20.99 years; 50.74% were men, and 49.26% were women. The WWI ranges for the first, second, third, and fourth quartiles were 8.04-10.45, 10.45-11.05, 11.05-11.64, and 11.64-14.14, respectively. Compared with participants in the lowest WWI quartile, those in the highest quartile were more likely to be male, older, excess alcohol consumers, or have CVD, lower education level, lower socioeconomic status, higher TG levels, higher BMI, higher total cholesterol, higher LDL, higher ALT, higher ALP, and higher serum uric acid.

**Table 1:** Weighted characteristics of the study population based on controlled attenuated parameter (CAP) and median liver stiffness measurement (LSM).

Weight-adjusted-waist index (WWI)	Q1 N=1,899 (8.04-10.44)	Q2 N=1,898 (10.44-11.05)	Q3 N=1,898 (11.05-11.64)	Q4 N=1,899 (11.64-14.14)	*P*-value
Age (years), (%)					<0.001
18-39	1,263 (67%)	693 (37%)	433 (23%)	266 (14%)	
40-59	184 (9.7%)	444 (23%)	775 (41%)	1,098 (58%)	
≥60	452 (24%)	761 (40%)	690 (36%)	535 (28%)	
Sex, (%)					<0.001
Male	754 (40%)	857 (45%)	963 (51%)	1,279 (67%)	
Female	1,145 (60%)	1,041 (55%)	935 (49%)	620 (33%)	
Race, (%)					<0.001
Mexican American	155 (8.2%)	227 (12%)	293 (15%)	256 (13%)	
Other Hispanic	162 (8.5%)	204 (11%)	202 (11%)	226 (12%)	
Non-Hispanic White	576 (30%)	603 (32%)	642 (34%)	785 (41%)	
Non-Hispanic Black	632 (33%)	487 (26%)	450 (24%)	391 (21%)	
Other Races	374 (20%)	377 (20%)	311 (16%)	241 (13%)	
Education level, (%)					<0.001
Less than high school	182 (9.6%)	284 (15%)	392 (21%)	435 (23%)	
High school or above high school	1,460 (77%)	1,541 (81%)	1,471 (78%)	1,439 (76%)	
Others	257 (14%)	73 (3.8%)	35 (1.8%)	25 (1.3%)	
BMI (kg/m^2^), (%)					<0.001
Normal weight	1,061 (56%)	522 (28%)	321 (17%)	169 (8.9%)	
Overweight	243 (13%)	674 (36%)	930 (49%)	1,246 (66%)	
Obese	594 (31%)	698 (37%)	644 (34%)	482 (25%)	
Diabetes, (%)					<0.001
Yes	61 (0.8%)	239 (3.1%)	436 (5.7%)	681 (8.9%)	
No	1,838 (24.2%)	1,659 (21.8%)	1,462 (19.3%)	1,218 (16.3%)	
Hypertension, (%)					<0.001
Yes	267 (14%)	581 (31%)	806 (42%)	1,038 (55%)	
No	1,632 (86%)	1,317 (69.02%)	1,091 (57.01%)	861 (45.02%)	
Smoking, (%)					<0.001
Yes	1,307 (69%)	1,160 (61%)	1,067 (56%)	1,089 (57%)	
No	592 (31%)	738 (39%)	831 (44%)	810 (43%)	
Excess Alcohol Consumption, (%)					<0.001
Yes	1,463 (88.08%)	1,378 (83.21%)	1,261 (77.08%)	1,073 (68.08%)	
No	198 (11.92%)	278 (16.79%)	375 (22.92%)	503 (31.92%)	
CVD, (%)					<0.001
Yes	61 (3.72%)	153 (8.37%)	203 (10.89%)	345 (18.36%)	
No	1,580 (96.28%)	1,675 (91.63%)	1,661 (89.11%)	1,534 (81.64%)	
Income to poverty ratio	2.40 (1.18, 4.66)	2.64 (1.27, 4.67)	2.31 (1.18, 4.16)	1.91 (1.11, 3.55)	<0.001
Laboratory features					
Total cholesterol (mmol/L)	172 (150, 198)	185 (161, 214)	187 (161, 216)	183 (156, 211)	<0.001
Triglyceride (mmol/L)	0.70 (0.52, 1.05)	0.96 (0.67, 1.49)	1.14 (0.80, 1.61)	1.24 (0.88, 1.70)	<0.001
LDL- cholesterol (mmol/L)	2.56 (2.07, 3.13)	2.79 (2.30, 3.41)	2.90 (2.25, 3.49)	2.69 (2.15, 3.34)	<0.001
HDL- cholesterol (mmol/L)	1.42 (1.19, 1.71)	1.29 (1.09, 1.60)	1.27 (1.06, 1.55)	1.27 (1.06, 1.53)	<0.001
ALT (IU/L)	15 (12, 22)	19 (13, 27)	19 (14, 27)	18 (13, 25)	<0.001
ALP (IU/L)	76 (56, 80)	71 (59, 86)	76 (64, 92)	82 (67, 99)	<0.001
AST (IU/L)	19 (16, 23)	19 (16, 24)	19 (16, 24)	19 (16, 23)	0.013
LSM (kPa)	4.70 (4.00, 5.70)	4.80 (4.00, 5.90)	5.10 (4.10, 6.38)	5.30 (4.30, 6.90)	<0.001
CAP (dB/m)	215 (190, 248)	257 (218, 297)	278 (238, 318)	295 (253, 336)	<0.001
Serum uric acid (μmol/L)	297 (244, 351)	315 (256, 381)	321 (262, 381)	321 (262, 381)	<0.001

Mean and interquartile range for continuous variables: P value was calculated by weighted linear regression model.

% for categorical variables: P value was calculated by weighted chi-square test.

BMI, body mass index; LDL- cholesterol, low-Density Lipoprotein Cholesterol; HDL- cholesterol, high-Density Lipoprotein Cholesterol; ALT, alanine transaminase; ALP, alkaline phosphatase; AST, aspartate aminotransferase; LSM, liver stiffness measure; CAP, controlled attenuation parameter; CVD, cardiovascular disease; WWI, weight-adjusted-waist index.

### 3.2 Association between the weight-adjusted-waist index and hepatic steatosis (CAP)

We first estimated the association between WWI and the severity of liver fibrosis without adjusting for any covariates. Higher WWI was associated with a higher grade of hepatic steatosis. After full adjustment (see Methods), each unit with a higher WWI score was found to be associated with 7.60 dB/m increased units of CAP [β=7.60, 95% CI (4.42, 10.78), P<0.001]. Sensitivity analysis was conducted after treating the WWI as a categorical variable (quartile). In the fully adjusted model, compared with the lowest WWI quartile (first quartile), the adjusted β for participants in the second quartile, third quartile, and fourth quartile were 6.51, 11.03, and 14.06, respectively (**Table 2**).

**Table 2:** The association between WWI with CAP and LSM.

Weight-adjusted-waist index	Crude model (Model 1) ^a^	Minimally adjusted model (Model 2) ^b^	Fully adjusted model (Model 3) ^c^
CAP β (95% CI) ^d^	31.21 (29.77, 32.65)<0.0001	36.97 (35.26, 38.68)<0.0001	7.60 (4.42, 10.78)<0.0001
WWI group			
Quartile 1	0	0	0
Quartile 2	37.55 (33.98, 41.13)<0.0001	39.48 (35.88, 43.09)<0.0001	6.51 (1.08, 11.94) 0.0188
Quartile 3	56.95 (53.37, 60.53)<0.0001	60.73 (56.92, 64.54)<0.0001	11.03 (4.89, 17.10) 0.0005
Quartile 4	72.75 (69.17, 76.33)<0.0001	81.61 (77.51, 85.72)<0.0001	14.06 (6.95, 21.17) 0.0001
*P* for trend	35.37 (33.69, 37.05)<0.0001	39.44 (37.49, 41.39)<0.0001	6.88 (3.50, 10.26)<0.0001
**LSM β (95% CI)**	0.72 (0.61, 0.84)<0.0001	0.90 (0.77, 1.04)<0.0001	0.03 (-0.26, 0.32) 0.8419
WWI group			
Quartile 1	0	0	0
Quartile 2	0.17 (-0.10, 0.45) 0.219	0.24 (-0.05, 0.53) 0.099	-0.44 (-0.94, 0.05) 0.0773
Quartile 3	0.94 (0.66, 1.22)<0.0001	1.06 (0.75, 1.36)<0.0001	-0.25 (-0.81, 0.31) 0.3825
Quartile 4	1.61 (1.33, 1.89)<0.0001	1.87 (1.54, 2.19)<0.0001	0.03 (-0.62, 0.67) 0.9314
*P* for trend	0.82 (0.69, 0.95)<0.0001	0.94 (0.79, 1.10)<0.0001	0.04 (-0.26, 0.35) 0.7835

In sensitivity analysis, Weight-adjusted-waist index was converted from a continuous variable to a categorical variable (quartile).

a Model 1: no covariates were adjusted.

b Model 2: adjusted for sex, age, and race.

c Model 3: adjusted for sex, age, race, education level, family income to poverty ratio, BMI, Diabetes, hypertension, smoking, drinking, family income to poverty ratio, Total calcium, Total cholesterol, Triglyceride, LDL- cholesterol, HDL- cholesterol, ALT, ALP, AST, Serum uric acid, drinking, and cardiovascular diseases.

d 95% CI: 95% confidence interval.

### 3.3 Association between the weight-adjusted-waist index and liver fibrosis (LSM)

As shown in **Table 2**, in the unadjusted model, each unit of higher WWI score was found to be associated with 0.72 kPa increased units of LSM [β=0.72, 95% CI (0.61, 0.84), P<0.0001]. However, after adjusting for all covariates, the relationship between WWI and LSM was not significant in Model 3 [β=0.03, 95% CI (-0.26, 0.32), P=0.84].

### 3.4 Subgroup analysis

We used stratified weighted multivariate regression analysis to investigate the association between WWI and CAP and LSM in different population settings, stratified by gender, age, BMI, T2D, hypertension, excess alcohol consumption, smoking, and CVD.

**Figure 2:**
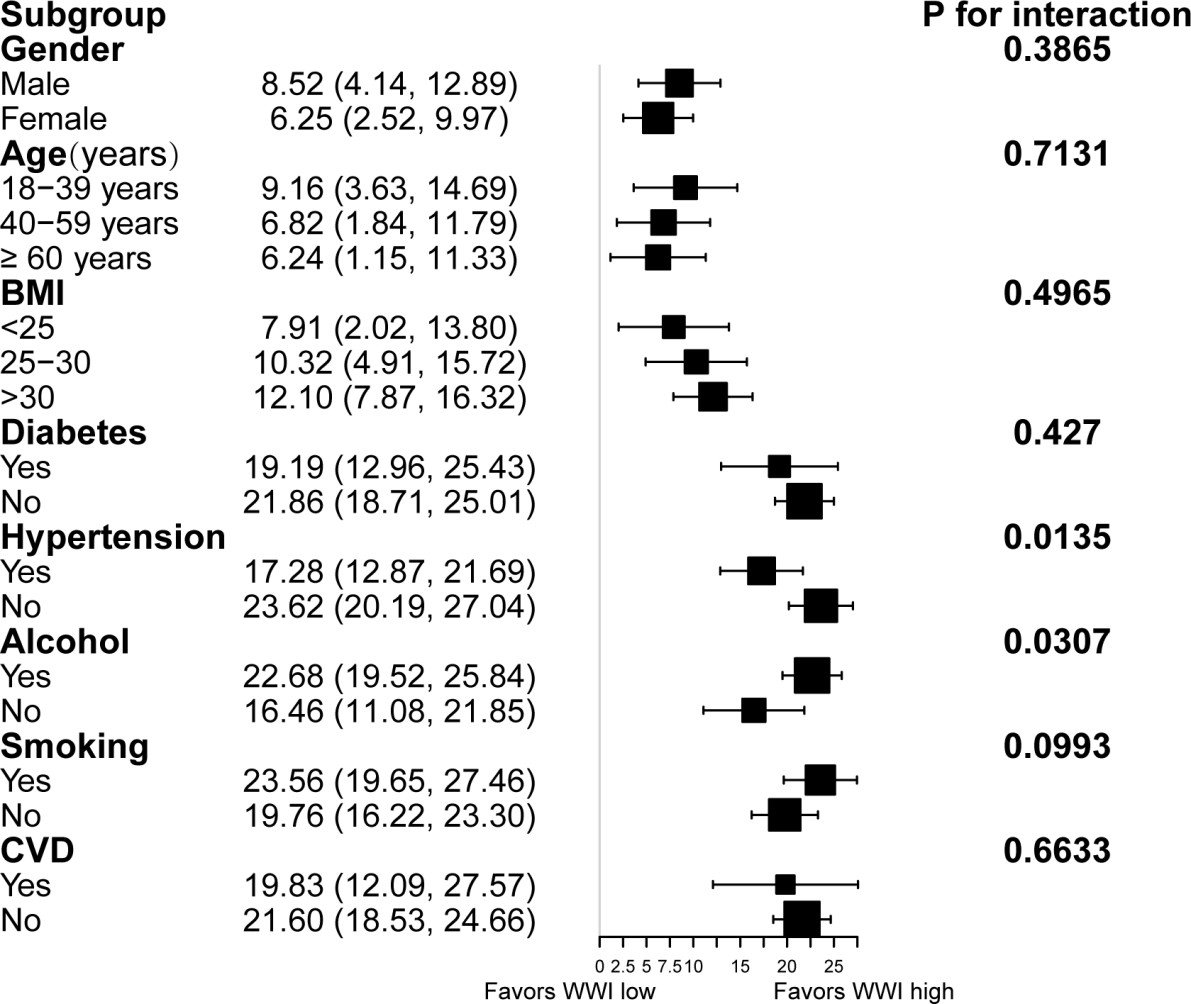
Subgroup analysis for the association between WWI and hepatic steatosis.

**Figure 3:**
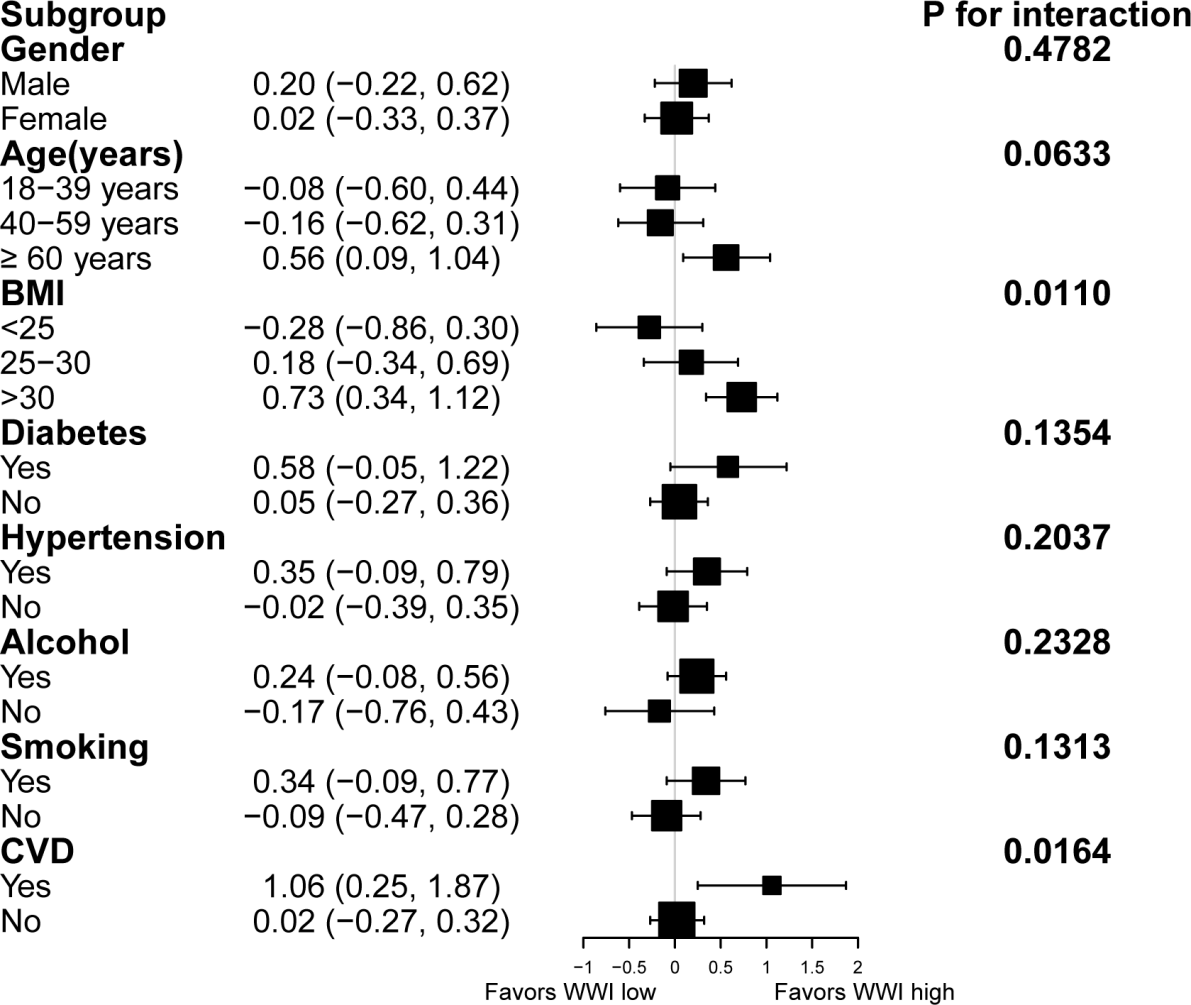
Subgroup analysis for the association between WWI and liver fibrosis.

As displayed in **Figure 2**, a stronger positive association between WWI and CAP was observed in participants with excess alcohol consumption and hypertension (P< 0.05). However, the correlation between WWI and CAP was similar in the population with different subgroups of gender, age, smoking, BMI, T2D, and CVD. Furthermore, A significant correlation between WWI and LSM was observed in participants with BMI>30 and experience CVD (**Figure 3**).

### 3.5 Non-linear relationship between WWI with hepatic steatosis and liver fibrosis

**Figure 4:**
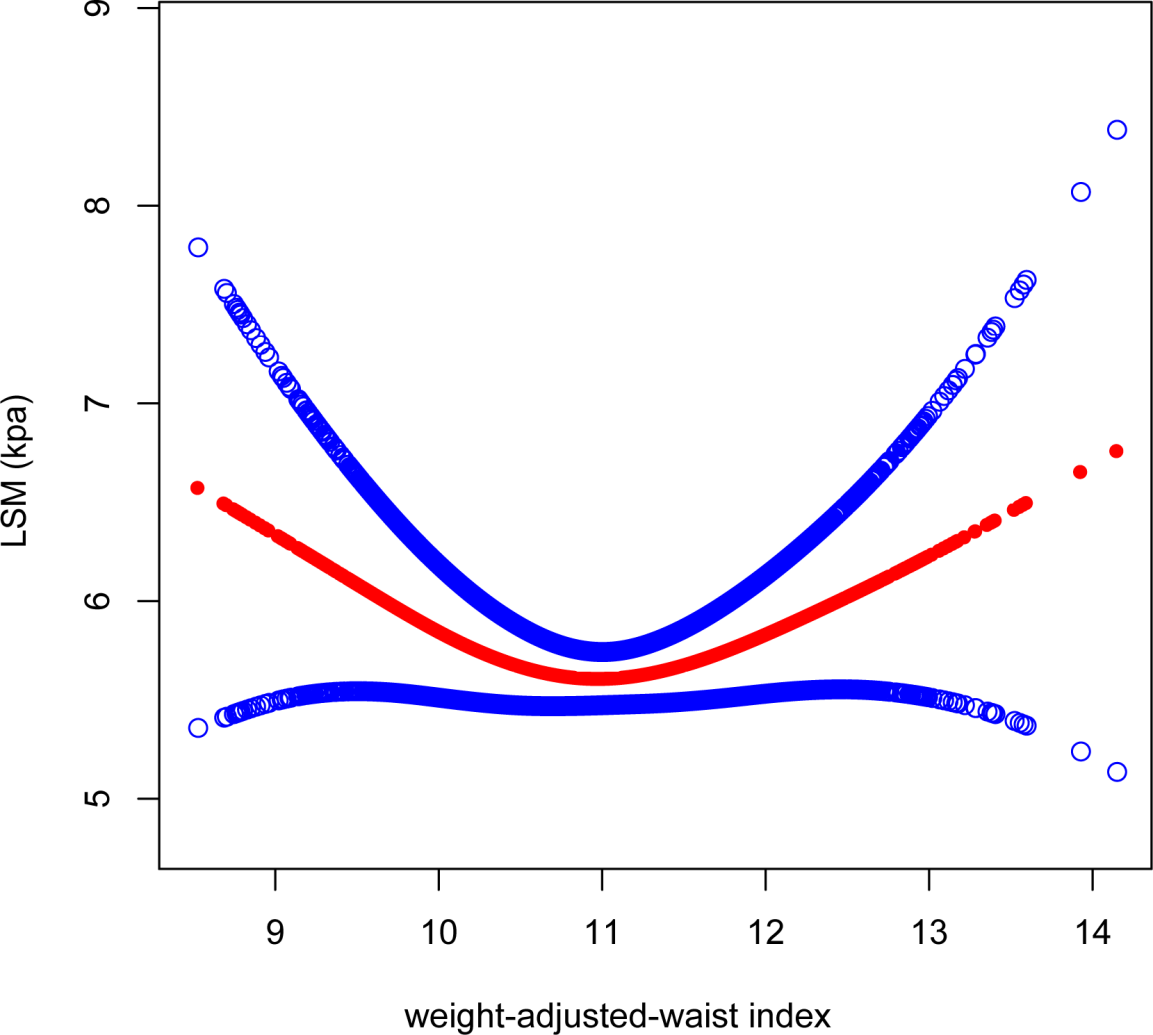
Smooth curve fitting for WWI and LSM. The solid red line represents the smooth curve fit between variables. Blue bands represent the 95% confidence interval from the fit. WWI, weight-adjusted-waist index; LSM, liver stiffness measurement.

After adjusting for all variables, a non-linear association between WWI and LSM levels was found (**Figure 4**). We observed a U-shaped relationship between the WWI and LSM (inflection point: 10.92) (**Table 3**). Specifically, LSM was negatively associated with WWI<10.92 [β=-0.57, 95% CI (-1.06, -0.08), P=0.022], and positively association with WWI >10.92 [β=0.44, 95% CI (0.05, 0.84), P=0.028].

**Table 3:** Threshold effect analysis of WWI on LSM and CAP using a two-piecewise linear regression model.

WWI	CAP Adjusted β (95% CI) P value	LSM Adjusted β (95% CI) P value
Fitting by the standard linear model	7.60 (4.42, 10.78)<0.0001	0.03 (-0.26, 0.32) 0.8419
Fitting by the two-piecewise linear model		
Inflection point	10.75	10.92
<K segment effect	12.98 (7.01, 18.95)<0.0001	-0.57 (-1.06, -0.08) 0.0219
>K segment effect	4.88 (0.80, 8.95) 0.0191	0.44 (0.05, 0.84) 0.0280
Log likelihood ratio	0.035	0.003

Adjusted for sex, age, race, education level, BMI, Diabetes, hypertension, smoking, drinking, family income to poverty ratio, Total calcium, Total cholesterol, Triglyceride, LDL- cholesterol, HDL- cholesterol, ALT, ALP, AST, Serum uric acid, drinking, and cardiovascular diseases.

**Figure 5:**
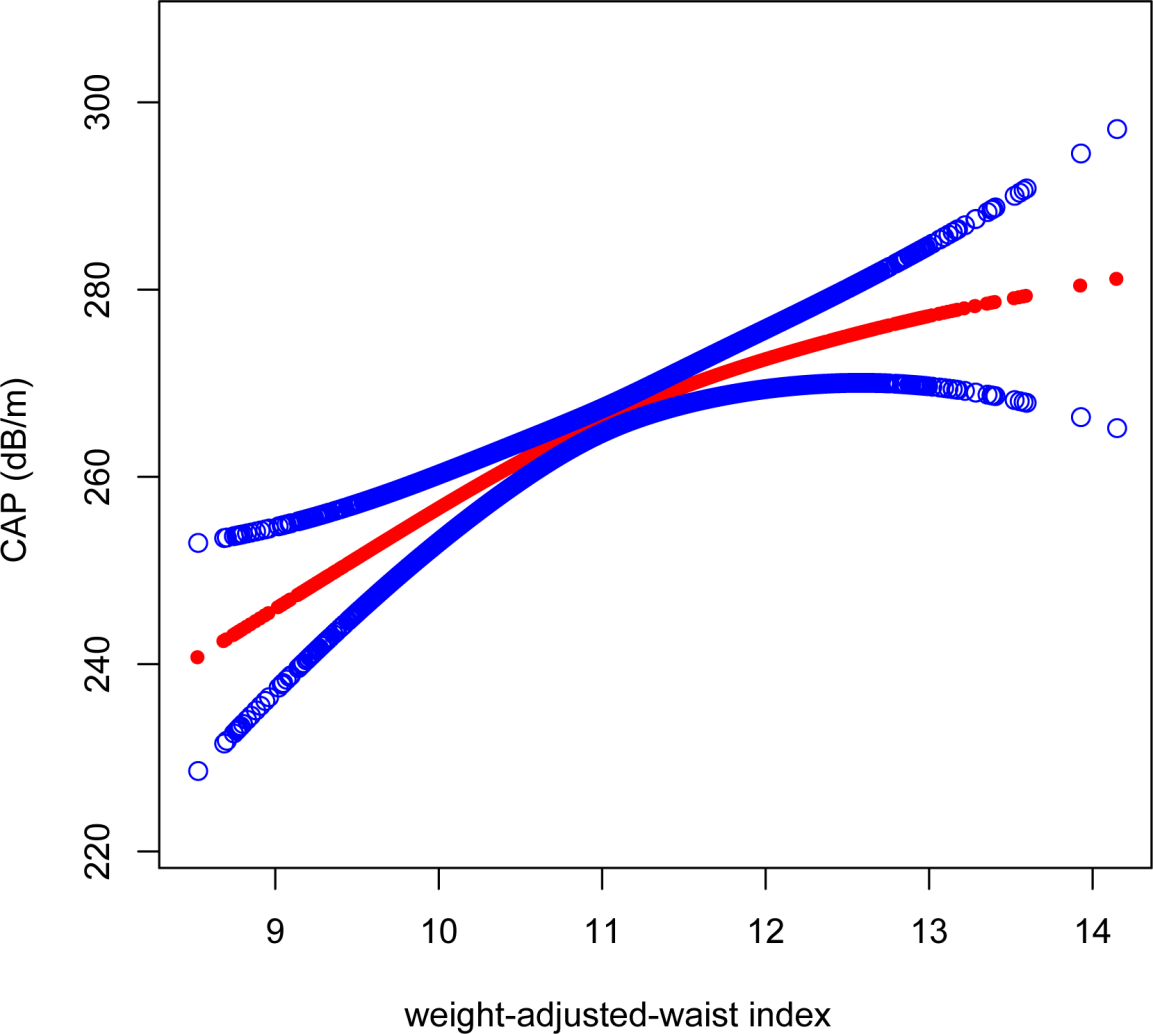
Smooth curve fitting for WWI and CAP. The solid red line represents the smooth curve fit between variables. Blue bands represent the 95% confidence interval from the fit. WWI, weight-adjusted-waist index; CAP, controlled attenuation parameter.

As with the linear association, we also observed a positive correlation between WWI and CAP when conducting the non-linear model. We found consistent positive association between WWI and CAP. Notably, the association was much stronger when WWI>10.75 [WWI>10.75: β=12.98, 95% CI (7.01, 18.95), P<0.0001]; WWI<10.75: [β=4.88, 95% CI (0.80, 8.95), P=0.02] (**Figure 5**).

## 4 Discussion

The present study aimed to evaluate the relationship between WWI and hepatic steatosis and liver fibrosis among civilians in the United States. In our cross-sectional study comprising 7,594 participants, we found a significant positive linear association between WWI and hepatic steatosis. Furthermore, we identified a U-shaped relationship between WWI and liver fibrosis (inflection point 10.92). By performing subgroup analyses, a stronger association between WWI and hepatic steatosis was demonstrated in participants with hypertensive disorders and excessive alcohol consumption. Additionally, we saw evidence of a strong correlation between WWI and liver fibrosis in participants with BMI>30 or CVD.

Accumulating evidence has supported the leading role of obesity in the pathogenesis of NAFLD. Given a strong link with dysregulated lipid metabolism, obesity not only contributes to the evolution of hepatic steatosis and inflammation, but also poses a threat to cardiovascular events and metabolic syndromes (20). Currently, BMI is widely used to determine the severity of obesity. However, obese patients, especially NAFLD patients, tend to demonstrate both fat accumulation and loss of skeletal muscle mass due to physical inactivity, which further increases the risk of hospitalization for NAFLD patients (21, 22). In addition, central obesity, specifically the accumulation of adipose in deep subcutaneous tissue, has been identified as the critical driver for NASH progression, insulin resistance, and cardiovascular events (23). Therefore, BMI, using total weight, may not accurately reflect the health status of obese individuals, particularly NAFLD patients. In contrast, WWI, calculated by normalizing WC with body weight, primarily reflects pure central obesity and can assess high fat mass and low muscle mass (24). For this study, we evaluated the association of WWI with estimated liver histology. We found a significant positive correlation between WWI and CAP. Additionally, we have identified a U-shaped association between WWI and LSM.

WWI was first used to better evaluate the morbidity and mortality of cardiovascular and metabolic diseases in the Korean population (25). Furthermore, trans-ethnic studies have revealed that WWI was positively associated with higher risks of hyperuricemia and multiple kinds of cardiovascular events, including heart failure, abdominal aortic calcification, and left ventricular hypertrophy (14, 26–29).

WWI was thought to represent the severity of central obesity rather than general obesity. As a sign of metabolic syndromes, a series of studies have suggested that central obesity is a risk factor for NAFLD, cardiovascular diseases, and other metabolic diseases for both obese and non-obese populations (30–32). Specifically, central obesity is characterized by the accumulation of abdominal adipose tissue, especially visceral adipose tissue. Mechanically, visceral adipose tissue could constantly secrete proinflammatory stimuli, which could further lead to systematic inflammation, metabolic disorders, and histological progression in the liver of obese patients. Moreover, during the expansion of visceral adipose tissue, proinflammatory macrophages in the adipose further contribute to immune-infiltration in the liver (33). In addition, some subtypes of adipose-derived ceramidases could desensitize insulin activity and contribute to insulin resistance (34). Our study has further highlighted and qualified the critical role central obesity in liver steatosis. In the future, WWI may be a liver fibrosis predictor.

This study has several limitations which should be mentioned. First, the cross-sectional study design did not allow us to determine any causal relationships. Second, the severity of hepatic steatosis and liver fibrosis in this study was determined using VCTE, which needs to be further validated in biopsy-proven cohorts. Furthermore, although the NHANES was conducted in the United States in a multi-ethnic adult population, our results may not reflect other geographic areas or ethnic groups.

### 4.1 Conclusion

In conclusion, our study found strong link between WWI and hepatic steatosis, and suggested that it may potentially be used as a simple anthropometric index to predict hepatic steatosis.

## Data availability statement

The original contributions presented in the study are included in the article/supplementary material. Further inquiries can be directed to the corresponding author.

## Author contributions

YS and YW contributed equally to the research’s conception and design. YS performed the study and wrote the draft. YS contributed to the acquisition and analysis of the data. MF and KZ conducted the statistical analysis. YS wrote the initial drafts of the manuscript. JW reviewed and revised the later drafts of the manuscript. All authors critically reviewed the manuscript, agreed to be fully accountable for ensuring the integrity and accuracy of the work, and read and approved the final manuscript. All authors contributed to the article and approved the submitted version.
